# How to Support Child Healthcare Nurses in Sweden to Promote Healthy Lifestyle Behaviors from the Start of Life

**DOI:** 10.3390/children8080696

**Published:** 2021-08-12

**Authors:** Matilda Ersson, Maria Henström, Gerd Almquist-Tangen, Kylie D. Hesketh, Christine Delisle Nyström

**Affiliations:** 1 Department of Biosciences and Nutrition, Karolinska Institutet, Neo, 141 83 Huddinge, Sweden; ersson.matilda@gmail.com (M.E.); maria.henstrom@ki.se (M.H.); 2 Department of Pediatrics, Institute of Clinical Sciences, The Sahlgrenska Academy, University of Gothenburg, 405 30 Gothenburg, Sweden; alta@halmstad.net; 3 Child Health Care Unit, Region Halland, 301 80 Halmstad, Sweden; 4 Institute for Physical Activity and Nutrition (IPAN), Deakin University, Geelong 3125, Australia; kylie.hesketh@deakin.edu.au

**Keywords:** child healthcare, food introduction, infant, physical activity, qualitative research, screen time, thematic analysis

## Abstract

Child healthcare (CHC) nurses have a key role in promoting and supporting healthy lifestyle behaviors from a young age. Thus, this study aims to investigate the perspectives of CHC nurses regarding discussing food introduction, physical activity/active play, and screen time with parents; explore facilitators and barriers influencing the discussion of healthy lifestyle behaviors with parents; and explore the perspectives of CHC nurses regarding a complementary program to promote healthy lifestyle behaviors from the start of life. A total of fifteen nurses participated in semi-structured interviews, which were recorded, transcribed verbatim, and analyzed using thematic analysis. There were four themes that were generated: parental needs; facilitators and barriers; parental groups; and future working methods. This study found that CHC nurses have seen an increase in the need for support among today’s parents. Time, the need to tailor information, and confidence to address sensitive topics were perceived as the largest barriers during daily work for the nurses. Furthermore, large variations in parental groups were found. Finally, the CHC nurses displayed a willingness and openness to change and develop current working methods using digital solutions. These solutions could possibly ease the workload and at the same time, support parents to create healthy lifestyle behaviors from the start of their child’s life.

## 1. Introduction

The first 1000 days of life (i.e., the time point from conception until 2 years of age) has been found to be a pivotal time in a child’s life, where lifestyle choices and behaviors can have lifelong consequences [1]. As lifestyle behaviors have been found to be established in infancy (i.e., 0–2 years) and track into early childhood [2,3], it is important to promote the development of healthy lifestyle behaviors. These are especially important to target early in life, as it gets progressively harder to break established behaviors with age [1].

To the best of our knowledge, only one health promotion intervention has been initiated in infancy in Sweden. The intervention targeted first time parents and their infant at 8–9 months of age through primary child healthcare (CHC) centers. Trained CHC nurses applied motivational interviewing embedded in the routine CHC services to promote healthy food and physical activity habits with the overall aim to prevent obesity. No statistically significant intervention effects post-intervention nor at the 1-year follow-up were found, and the authors attributed the lack of effect to an imperfect implementation of the intervention [4,5]. This study was initiated when the children were 8–9 months old, so it is therefore conceivable that it may have been too late, as children are introduced to food as early as 4–6 months of age. Thus, there is a need for scalable interventions starting earlier in infancy to explore whether age is an influencing factor.

Sweden’s CHC system is voluntary, free of charge, and reaches almost 99% of the approximately 115,000 children born each year [6]. CHC nurses meet the parents and children continuously from birth until the children turns five years old during individual health visits according to the intervals suggested by the Swedish National Healthcare Program [7], thus making CHC nurses a main point of contact for healthcare during a child’s first years of life. In addition to the individual health visits where the nurses measure and register the child’s growth and development, primary CHC are required to offer all parents the opportunity to participate in a parental group [8]. The three main goals for these meetings are (i) to increase parental knowledge, (ii) to create opportunities for parents to generate new contacts, and (iii) to create opportunities for the awareness of and impact on societal conditions [8]. However, how parents are invited to these sessions as well as the content and structure of these meetings are designed by each individual CHC center, allowing for regional and national differences in how and what information is provided to the parents. This was observed in two recent studies showing large variations in parental groups in terms of content, structure, and frequency [9,10].

Additionally, the Swedish National Board of Health and Welfare have identified that there is a need of more material, education, and working methods to promote healthy lifestyle behaviors within primary CHC [6]. They have also stated that the CHC system needs to develop and adjust their way of working in line with societal development as a way to meet current and future tasks within the field of public health. Therefore, this emphasizes the need for scalable interventions with the aim of improving lifestyle behaviors from the start of life while also aiming to minimize inequalities between regions and caregivers.

Developing new interventions can be costly, making it financially advantageous to implement and adapt existing promising interventions to new settings [11]. A promising intervention is the Melbourne Infant Feeding Activity and Nutrition Trial (INFANT), which was a multi-component intervention delivered through primary CHC targeting first-time parents and their infants in Victoria, Australia. The intervention has shown promising results with regard to television viewing and diet post-intervention when the infants were approximately 20 months old [12], with these results being sustained until five years of age [13]. The intervention, now delivered in a state-wide scale up, consists of four group sessions starting when the infants are approximately four months of age. Parents are recruited from first time parent groups and each group comprises between four and ten parents. Examples of topics covered in the group sessions include feeding practices, active play, and sedentary behavior [14]. These sessions are also complemented by a mobile app that includes reinforcing messages for parents that are adjusted for the child’s age and stage of development [14].

The similarities in free universal healthcare available to infants in Sweden and Australia make a program such as INFANT a promising intervention for the Swedish context. However, the program needs to be carefully adapted for the target population (i.e., parents and CHC nurses in Sweden) to ensure maximum cultural and contextual relevance. A recent qualitative study found that Swedish parents were positive towards a program similar to INFANT and that they felt that the information and support within the program could advantageously act as a complement to the current care provided by CHC [9]. Furthermore, CHC nurses have been identified as a profession that is well positioned in the role of promoting and supporting healthy lifestyle behaviors from a young age [15,16,17] and are thereby exceptionally suitable for the role of implementing such a program.

Therefore, the aims of this study are to (i) investigate the perspectives of CHC nurses regarding discussing food introduction, physical activity/active play, and screen time with Swedish parents; (ii) explore facilitators and barriers influencing the discussion of healthy lifestyle behaviors with parents of infants; and (iii) explore the perspectives of CHC nurses regarding a complementary program to promote healthy lifestyle behaviors from the start of life.

## 2. Materials and Methods

### 2.1. Participants and Recruitment

Participants were recruited through purposive sampling. There were three regional dietitians in two different regions in Sweden who were contacted: two in the region of Västra Götaland and one in the region of Jönköping. The dietitians recruited a total of 17 nurses from various CHC centers in their region who were interested in participating and provided us with their contact information. CHC nurses were recruited from both urban and rural locations as well high and low socioeconomic areas. The nurses were then sent the full study information as well as formal consent via email. They were all provided with the opportunity to ask questions and were requested to send their signed informed consent via mail if they were willing to participate. Once we received their informed consent, a telephone interview was scheduled at the nurse’s convenience. There were two nurses who chose not to participate after receiving the full study information; one nurse did not reply to the emails, and one nurse chose not to participate due to a lack of time. Thus, a total of 15 nurses participated in this study.

The inclusion criteria for this study were that the nurses had to be professionally active within a primary CHC center in the Jönköping region (population ~360,000) or the Västra Götaland region (population ~1.7 million), and there were no exclusion criteria. All CHC nurses in Sweden are registered nurses and have a specialization in healthcare for children and youth. All interviews were held between February–March 2021. The consolidated criteria for reporting qualitative studies (File S1) [18] was used in the reporting of this study. All participants provided both written and verbal consent before the interviews were initiated, and the study was approved by The Swedish Ethical Review Authority (2020-00814).

### 2.2. Semi-Structured Interviews

The interviews were semi-structured and performed once in Swedish over the phone, and only M.E. and the interviewee were present. No relationship was established between the interviewer and the interviewee before the commencement of the interviews. All participants received verbal introductory information explaining the aims of the study at the beginning of the interview. The interviewee was then asked for verbal consent and confirmation of permission to record the interview (audio). Thereafter, the interview continued with the 15 core questions (File S2). Additionally, before part two of the interview, the participants were told about the cooperation with Australia, and the INFANT program was briefly described to them as an example of how a program supporting parents to promote healthy lifestyle behaviors could potentially look like. The core questions for the interviews were constructed and were critically discussed by the whole research team (all female), with extensive experience with childhood obesity prevention. Of the researchers, three are nutritionists (M.E., M.H., C.D.N.), one is a child healthcare nurse (G.A.T.), and K.D.H. is a public health researcher. All of the researchers have a PhD, except for M.E., who has a MSc. The background of the researchers has been made clear, as data interpretation can be influenced by this.

The interview guide was piloted with two CHC nurses before the commencement of the main interviews to ensure the feasibility and quality of the core questions. These interviews gave insight into areas in need of improvement and resulted in small amendments added to the interview guide to expand and deepen the interviews. All participants were asked the 15 core questions with follow-up questions adapted to the provided answers in a semi-structured manner.

All of the interviews were audio recorded using an Olympus VN-405PC dictaphone and were transcribed verbatim using a professional transcribing service. The transcripts were not returned to the interviewees for comments or corrections. The duration of the interviews varied from 34–78 min, with a mean recording time of 49.9 min. Reflexive notes were written during and directly after the interview to capture impressions and contextual information.

### 2.3. Data Analysis

The interview transcripts were analyzed using thematic analysis [19,20]. A realist method with an inductive analysis and a semantic approach were used to find the repeated patterns and their meaning across cases. These methods focus on the experiences and the reality of the participants without trying to fit the generated codes into a pre-existing coding frame [19]. These methods also help to identify codes that generate themes, which captures the surface meaning of the answers without examining the underlying meanings of the participants’ responses or anything beyond the actual answer from the participant. All of the interview transcripts were read and re-read by M.E. and C.D.N. The interview transcripts were then coded by M.E., who was supported by continuous discussions on the perceived codes together with C.D.N. and M.H. The perceived codes were sorted in Microsoft Excel (Microsoft 365 version 2103) by M.E., and themes were created. As the interviews were conducted in Swedish, the following analysis was performed in the original language, and the quotes presented in this article were carefully translated into English from the original transcripts. Statistical analysis of the descriptive characteristics of the participants was performed with Microsoft Excel (Microsoft 365 version 2103).

## 3. Results

**Figure 1:**
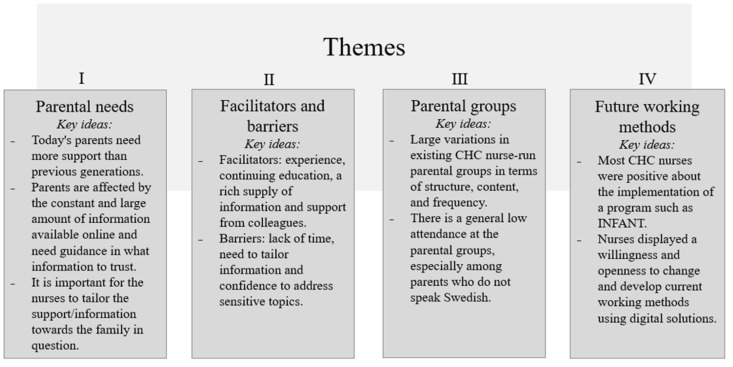
Themes and key ideas generated in the thematic analysis.

The participants were all female with a mean age of 50.2 ± 7.2 years and had 11.7 ± 6.4 years of working experience within primary CHC. There were four themes that were generated from the thematic analysis: (i) parental needs, (ii) facilitators and barriers, (iii) parental groups, and (iv) future working methods (Figure 1).

### 3.1. Theme 1: Parental Needs

When asked which kind of parent might need more information and support, the majority of the CHC nurses reported that recent first-time parents need the most support and information in comparison to parents with prior children. Several CHC nurses also mentioned the pronounced change that has taken place in recent years. It was perceived that today’s parents need more support in general, compared to previous generations.

*“I feel that you need to give more support to the parents today. They are more insecure about their parenting role than I experienced when I started as a CHC nurse 20 years ago.”*

(Nurse 10)

The change of parents needing more support over the years was also thought to be due to the social conditions of today, such as families living further away from their relatives and not having as broad a social network among colleagues and neighbors today compared to a few years ago. The lack of social networks was a recurring topic when discussing about the needs of today’s parents. Subsequently, as social networks are decreasing, the role of CHC nurses is becoming a more important role in parents’ lives.

*“I think it’s partly because there is so much information circulating. What to believe, what’s right and what’s wrong? You [the parents] may not really have the network around you that you had 20 years ago, so maybe you had grandparents closer than you have today. So to speak, the network.”*

(Nurse 10)

The information found online by the parents was primarily from social media or from discussion forums, and several CHC nurses mentioned how parents needed guidance and support regarding the validity of the information they found. Furthermore, the nurses also perceived that some parents got stressed by the feeling of always having to keep up-to-date online.

*“But it can also be way too much information [online], so many times, I have to help them to sort the information that is available…//Because it’s very difficult for them to decide. They get quite confused by everything that exists.”*

(Nurse 9)

It was also perceived by the nurses that parents tended to be impatient when they had questions. Some parents collected questions between individual meetings and brought them to the visit, while others called their nurse for guidance directly. However, the majority of parents seemed to go online to find the answers right away. The nurses also expressed that even if the parents found the answer online, they still wanted to confirm the answer with them.

*“You [the parents] are used to finding information and have an exchange of information via social media, you are looking for answers yourself, and you have a need to get answers fairly immediately. You do not dare to wait. You have to know now.”*

(Nurse 6)

In order to support parents in avoiding questionable information online, the CHC nurses recommended verified and trusted websites such as the Swedish Food Agency and the Regional Online “care guide” (1177.se). The questions that were most asked by the parents at the individual meetings were regarding breastfeeding and food introduction. There was a clear increase in the number of questions and the need for support during the first weeks after birth but also between 6–10 months. Food introduction was described as a tough and challenging period for many parents. According to the nurses, parents could often describe it as stressful, and many would show fear of making mistakes.

*“First of all, I do not experience that there are so many questions when you have these tiny tasting portions, and you start testing by the teaspoon, then it goes pretty well for many. But then when they start to get a little bigger…at that time, it’s a little worrisome what to give their children to eat, what consistencies the food should have, when to introduce water and drinking as a complement and…Yes, there is a lot of worry and a lot of questions.”*

(Nurse 15)

Another element that came across when discussing how to meet and how to communicate with parents was the importance of adjusting the way of working towards the parents in question. This was mainly regarding factors such as language, culture, and socioeconomic position. For some parents, the information provided had to be in simple Swedish or focused around the most important subjects in order to enable the parents to take in all of the information that was provided. For families that could not speak Swedish, the use of interpreters over the phone or through pictures was a common method to ensure that the information that was provided was understood. For some families, the information had to be adjusted to their lifestyle and culture, and some nurses mentioned the importance of considering different cultural aspects such as ways of eating and what foods were common in a particular culture when discussing feeding practices or recommendations.

*“And then I went home [to the family] and made a food observation and realized that yeah, they come from a country where you do not sit at a dining table; you sit on the floor and eat directly from the bowls. We have a lot to learn, how it looks like across our cultural boundaries.”*

(Nurse 10)

### 3.2. Theme 2: Facilitators and Barriers

When asked what made the nurses feel secure and safe in giving advice and information regarding subjects such as feeding practices, food introduction, physical activity/active play, and screen time, the majority of the nurses expressed that experience, continuing education, a rich supply of information and material as well as support from colleagues where the main facilitators. Most nurses stated that they felt secure in giving advice and information regarding feeding practices, food introduction, and physical activity/active play early in life. The nurses felt secure because they felt that they had good support from their regional dieticians regarding food introduction and could turn to them if they had questions. Furthermore, they also felt more comfortable if they had access to information and material regarding the subject. However, almost all of the CHC nurses described that they did not feel comfortable in giving advice and information about screen time. Screen time was considered to be a sensitive subject with parents, and it was a difficult subject to discuss. Moreover, the nurses considered screen time to be a new subject and had very little material to support discussions with parents. Many nurses expressed a wish for more information regarding screen time to enable a conversation around the topic earlier in the child’s life.

*“It’s a really sensitive topic [screen time]…and because everyone probably has very different opinions about it, with screen and children…and that it is very new and that there is not so much research done.//We need more information [regarding screen time]. Eh, we need good information that you can hand out, and how to talk to parents about this [screen time]. It is so early that they [the children] start with screens.”*

(Nurse 11)

Furthermore, if the CHC nurses discussed screen time with the parents, it was usually first mentioned when the child was 18 months of age or older. This was due to more information being available at that time point and because some parents were asked to fill in a questionnaire regarding screen time during that time. If screen time was discussed before the age of 18 months, the nurses said it was because there was a natural segue into the conversation, e.g., the television being turned on during home visits or the parents letting their child play or watch something on their phone during visits at the CHC center.

*“We ask at two and a half years, how much time… how much screen time they have. This is probably the first time we bring it up as a standard...//I usually bring it up at the home visit at eight months, as often there is a TV on when you arrive. Eh, and then you get a natural entrance around it.”*

(Nurse 10)

One of the main barriers that came across during the interviews was the perception of a constant lack of time. Time was an important factor when discussing the individual meetings between CHC nurses and parents. Furthermore, some nurses expressed a certain time pressure of managing everything that had to be completed during the individual meetings.

*“The most common time is 30 minutes [for individual meetings]. And then you have to weigh or take…check the growth. You might have to give some information. So, sometimes it is very stressful.”*

(Nurse 4)

The time pressure during individual meetings was considered to be especially challenging when the meeting was adjacent to a doctors’ appointment, as the time for the doctor’s appointment was taken from the allocated minutes for the individual meeting. This could mean that information regarding food introduction that was supposed to be handed out at six months had to be given at another time.

*“Then we have a doctor’s visit, so then unfortunately, you do not have time to talk quite as much about this with food and living habits, but of course, you check that the parents do not have questions or are stuck somewhere or so.”*

(Nurse 15)

Many of the nurses stated that a large part of the time within the individual meetings was spent answering questions, which was thought to be an important, and they prioritized this as part of their job. A few CHC nurses felt that they had enough time to answer all of the parent’s questions, while the majority of the nurses mentioned that they often did not have time to answer all of the questions during one meeting and that they had to reschedule the parents for a new individual meeting or a meeting over the phone.

*“And if it’s so that you see that it is a lot [of questions], that this… now it will be tight with time, and I do not have that time, then you can book a new meeting next week as well; then you get an extra meeting. It’s no problem to do it. I think I have time for that. Sometimes, you have to squeeze in a little extra, or it goes over, and then you get less coffee break there and so, but I still think it’s important that they [the questions] are asked.”*

(Nurse 5)

As answering questions was considered to be an important part of their job, many nurses stated that their own plans for the meeting may have had to be de-prioritized. This was also something that could happen if the family in question had a trauma or a significant life event that had to be prioritized.

*“And some parents have written them [the questions] in their mobile, and some visits can just take like…all these questions take time. And then you have to abandon the things that you really intended to convey at that time. You have to bring that up on another occasion.”*

(Nurse 4)

Time was also perceived as a barrier when trying to keep up-to-date with the national handbook for CHC nurses or other new information within the field. Furthermore, when discussing the implementation of new working methods, time was the primarily factor that had to be taken into account.

*“If you read the national handbook [Rikshandboken] and the care guide [1177.se], there are very good things in there. You will be surprised, you will be completely… We do not have time to read so much, but when…when you are there reading, you feel, oh, everything is written here.”*

(Nurse 12)

It was perceived that there was a lack of knowledge when it came to other cultures and ways of living different then their own. CHC nurses also found language to be a large barrier in some interactions with parents. This was the most apparent in group sessions, but it was also apparent in individual meetings. The use of interpreters was considered both to be a facilitator and a barrier. Several nurses expressed that the use of interpreters is essential when communicating with parents who do not speak Swedish. However, it could also be problematic, as the communication had an intermediary that might have affected the information that was delivered.

*“For example, if you sit with an interpreter and then you think to yourself, did it come out right, all that I wanted to convey? Or did mom and dad understand?...There are those aspects as well.”*

(Nurse 2)

### 3.3. Theme 3: Parental Groups

When discussing parental groups with the CHC nurses, it was clear that the working methods and the execution of parental groups differed between the two regions but also between CHC centers within a region. Most nurses explained that only first-time parents were invited to the parental groups, while some nurses stated that all families were welcome. Further differences included the number of meetings that were offered. These varied from 4–9 occasions, and one nurse explained that they had not offered any parental groups in the last five years. Most nurses reported a general low attendance at the parental groups, and this was thought to be due to external factors such as low interest and lack of time. Additionally, there was a clear perception among the nurses that parents who did not speak Swedish generally did not attend the parental groups, which was thought to be due to the language barrier and the challenge of using interpreters in a joint space. The nurses who reported a high attendance for the parental groups also emphasized the amount and effort they put into inviting the parents.

*“We really cared about that many would want to come…would come to these meetings, so we called and invited, and we sent out letters.”*

(Nurse 8)

To counteract the low attendance at the parental groups, some nurses explained how they had incorporated baby massage as a method to reach out to parents who do not speak Swedish. The nurses pointed out that the parents who did not come to the parental groups needed to receive the information during the individual meetings instead. Several nurses also mentioned how they had tried to change or update the way that the parental groups are implemented.

*“Because it has been a good way [baby massage] to get those who do not have Swedish [as a first language]. Then they have been able to participate and massage. Because it is always when you have a group…eh…that it can be difficult with the language. And then we do not reach those who do not speak Swedish very well.”*

(Nurse 7)

The COVID-19 pandemic has affected the ability to provide parental groups. Most nurses described how there has been a will to find solutions to maintain the parental groups during the pandemic, but that it has been difficult. Some explained how they have offered outdoor walks with smaller groups, some have tried to reach out through social media to create a community, some have tried digital meetings, and some did not offer any alternatives at all.

*“During COVID-19 [pandemic], there have been some pram walks. Now it is the parents themselves who find each other via the internet and arrange meetings and go out for walks and so on together.”*

(Nurse 12)

When asking the nurses if they had received any education or training in leading parental groups, some nurses stated that they have had training during their nursing education, and some have received further training through their CHC center or from the region. However, some could not recall any specific training or education regarding how to lead parental groups. The majority of the nurses were positive towards the idea of continuing education for leading parental groups.

*“So, yes, you can never have too much knowledge, and you always need to be updated. You always need to bring your knowledge back to life, so to speak.”*

(Nurse 15)

### 3.4. Theme 4: Future Working Methods

When introduced to the INFANT program, all of the nurses expressed a positive interest regarding such a program between the ages of 2 and 18 months. Some nurses expressed that a program similar to the one presented were in line with their own wishes for a change and development in contemporary parental groups.

*“I look forward to this [a change from todays practice], and I…I think this is the way to go for our parental groups. It feels great and especially because we have also talked about that, we have to redo our parental groups, so this feels…I think this will be great, and I think it will result in something good that you feel safe with or that this is what we want to talk about, this is what we want to get out.”*

(Nurse 10)

Some nurses expressed that their expectation and hope for this kind of program was also that it would contribute to more equal and similar working methods within primary CHC nationally, which several nurses wished for.

*“It would have been great, that you get support with materials, and that…Yes, but also that everyone does the same. Especially for parental education, I think it looks very, very different [parental groups across Sweden].”*

(Nurse 4)

The nurses were also cautiously positive regarding the possibility of using digital solutions for parental groups. Most nurses could see the benefits in offering parental groups digitally, such as reaching out to parents who do not feel comfortable in groups, parents who live far away from the CHC center, or younger parents who are used to communicating online. Some nurses stated that digital solutions are the future and that it was important to keep up-to-date with them.

*“I think that this is the future in a way actually [digital solutions]. COVID-19 [pandemic] allows us to rethink and think new. Eh…and we might have a higher attendance if we have it digitally, that you do not feel the need of getting yourself and the child ready and go somewhere, so you can just log in.”*

(Nurse 6)

However, some nurses also expressed the importance of in-person parental groups as well as the perception that communication might be hindered when not meeting physically and that it would be hard to read the body language and the mood of the parents though a screen.

*“I do not think that we should just do it digitally, but I think it is better that you get together. Or that you do both of them possibly, so those who might live far away and do not get here or something like that…”*

(Nurse 5)

The majority of the nurses were positive to the idea of introducing a smartphone app containing information such as feeding, food introduction, active play, and screen time to parents. Most nurses saw the app as a possibility to convey evidence-based information to the parents that they could stand behind and that it would be positive for the parents to have access to such information in-between individual meetings.

*“I think it’s good [an app] because then they get the information that we stand for. Eh, and where we know it’s ensured anyway, the information that’s there. So absolutely, it’s great, because otherwise… they still use their mobile phones, and they go out and surf on other websites.*“

(Nurse 1)

Furthermore, most nurses thought that an app would be an easily accessible tool that everyone could take part in, as almost all parents use a smartphone today. When asked for what the nurses’ thought were important components and content for the app, most of the nurses requested an easy-to-use platform including different languages, pictures or short movies for communication, concrete tips and tricks for the parents regarding the aforementioned topics, and evidence-based information aimed at both parents and nurses. It was also considered important that the app was attractive with a positive approach and an appealing layout so that the parents would want to use it.

*“Different languages are probably great.//that they [the parents] have the app in their own language, would be very…very fantastic.”*

(Nurse 1)

## 4. Discussion

The aim of this study was to investigate the perspectives of CHC nurses regarding discussing food introduction, physical activity/active play, and screen time with Swedish parents as well as to explore facilitators and barriers influencing the discussion of healthy lifestyle behaviors with parents of infants. The study also aimed to explore the perspectives of CHC nurses regarding a complementary program to promote healthy lifestyle behaviors from the start of life. There were four themes that were generated from the thematic analysis: (i) parental needs, (ii) facilitators and barriers, (iii) parental groups, and (iv) future working methods. The different themes highlight how the role of CHC nurses has developed over time and that the nurses have had to adjust their way of working as the parents of today face new challenges and express a need for other types of support as society develops and social conditions change. The nurses expressed how their access to information and material influenced how confident they felt in their profession, and the need for reliable and up-to-date information was evident throughout the interviews. It was also noticeable how important it was for the nurses to adjust their communication and their way of working towards the family in question. Furthermore, the interviews also displayed a willingness and openness among the nurses to change and develop current working methods.

When discussing the perceived increase in parental needs for support over the last few years, the reduction or lack of social networks for the parents was a common topic that came across during the interviews. The importance of a social network and support was expressed in a study looking at facilitating and inhibiting factors in the transition to parenthood, where the interviewed parents stated that family, friends, and acquaintances were an important part of a safety net, where they could get support and help [21]. Moreover, the perceived change is also supported by a report from the Swedish National Board of Health and Welfare, where representatives from different regions express a trend with an increased need for access to CHC and that today’s parents have more questions and are more worried about their child than previous generations [6]. Additionally, a review looking at the importance of social relationships also stated that social disconnection is becoming increasingly common in Western cultures [22]. These notions reinforce the idea that a lack of a social network for some parents necessitates these parents needing to source that support elsewhere, in this case from the CHC nurses, increasing their workload.

A further observation by nurses of parents’ information seeking was that parents had a need to get answers to their questions immediately, which often led to the parents searching for answers online. This perception is coherent with the opinion of American mothers who stated that they appreciated the possibility of having access to unlimited information online at any time [23]. Furthermore, a systematic review and meta-synthesis including 12 studies also reported that new parents indicated a need for a telephone helpline available day and night when needing guidance by a midwife [24]. However, the nurses in the present study also perceived that the constant access to information online was a stressor for some parents, and the nurses explained that they had to support and guide some of the parents to sift through all of the information that they had found. This phenomenon was also expressed among the American mothers, as they described that the amount of information could become overwhelming, especially when there were multiple viewpoints on a specific topic [23]. Thus, this demonstrates the importance of providing a tool similar to the app described earlier. All of the nurses in the present study were positive to a smartphone app containing evidence-based information that the parents could use between individual meetings. A recent study interviewing Swedish parents found that the majority of the parents appreciated the usage of smartphone apps and that they were positive to an app provided by primary CHC within a program such as INFANT [9]. Providing an app though CHC might minimize the parental need to search for additional information online, which may reduce the risk of the parents receiving misleading and contradictory information from unreliable online sources. Furthermore, it also allows the nurses to have insight into what information the parents receive and have access to.

Alongside the change in information seeking and the information environment, nurses identified three key barriers to their own delivery of information to parents: their confidence, the need for individual (particularly cultural) tailoring of information, and time constraints. While confident in nutrition and active play, the majority of the nurses expressed that they did not feel secure in conveying information regarding screen time and that there was a need for more material and information to support nurses in broaching this sensitive subject. Screen time was also a distinct topic in the study by Laws et al., where nurses stated that they wanted more information on how to talk about limiting screen time with the parents, as this was considered a sensitive topic where the fear of offending the parents caused the nurses to avoid the subject [15]. Henström et al. also reported that parents regarded screen time as a sensitive subject [9]. In addition, both nurses in the present study as well as the nurses in the study by Laws et al. [15] emphasized the importance of conveying information about sensitive subjects without judgement in a way that is perceived as positive by the parents. It is incredibly important to provide CHC nurses with more material and/or information to enable screen time discussions with parents, as screen time habits formed at an early age may track overtime [25].

The importance of adjusting information towards the family’s lifestyle and culture was mentioned by the nurses, but at the same time, several nurses mentioned the lack of knowledge about other cultures other than their own. A study by Laws et al. also reported how maternal and child health nurses expressed a desire to know more about how to tailor interactions and recommendations to different cultures [15]. Furthermore, a recent integrative review looking at migrant family experiences also highlighted “the need of culturally safe interventions and care environments”, as migrant families reported that the care provided within maternal- and child- healthcare were not always culturally appropriate [26]. Thus, it is important to find working methods that can be adapted to different cultures. Digital working methods and smartphone apps with culturally adapted content might facilitate this.

The nurses in the present study stated that time was one of the main barriers within their daily routines to providing parents with information. Supporting this, representatives from CHC report that “there is hardly any telephone availability or opportunity for unplanned visits or conversations with a CHC nurse, in addition to the booked visits” [6]. The lack of time during individual meetings was also frequently mentioned in studies looking at the perceptions of nurses when talking about or addressing overweight and obesity in children [15,27,28], which may also incorporate discussion of obesity-protective behaviors that were found in this study. Together, this highlights the importance of finding time efficient working methods that can facilitate the dissemination of key information to parents. This could include a greater focus on group sessions where information can be disseminated to multiple parents simultaneously and the provision of evidence-based information endorsed by nurses through an online portal or app. These strategies, discussed in the context of the INFANT program, were appealing to nurses.

While the parent groups are a feature of CHC practice, when nurses were asked to describe how the parental groups were held, it was clear that there were large differences in the attendance and the execution of the parental groups between regions and also between CHC centers within a region. The perceived differences in the execution of parental groups were in line with the perception of Swedish parents [9,10]. Furthermore, the nurses reported a generally low attendance at parental groups even before the COVID-19 pandemic, which is in line with the findings of Karlström and Rising-Holmström [10]. Difficulties in engaging parents was a particular issue for those from non-Swedish speaking backgrounds. As a higher prevalence of being overweight and low levels of physical activity have been observed in children of immigrant parents [29], it is of the utmost importance to ensure that parents who do not speak Swedish are given the same access to information as parents who speak Swedish. Thus, new ways of delivering parental groups and engaging parents from all backgrounds are needed to improve attendance and to ensure consistency and equality between CHC centers. One nurse suggested that it would be great if there was a possibility to create online parental groups for parents speaking the same language. A structured program such as INFANT could enable this possibility, allowing for parental groups in different languages to be feasible.

One of the strengths of the study was the strong methodology including the piloting of the interview guide with two CHC nurses before the commencement of the main interviews. This allowed for feedback from the target population, thereby enabling a more suitable and targeted interview guide. Additionally, during thematic analysis, saturation was reached, where no new codes or themes were found. Purposeful sampling was chosen for this study, as it enabled the selection of participants and areas that are be representative of the target population and thereby increase the chance of the research questions being adequately answered [30]. Furthermore, there is a possibility that the nurses who agreed to participate in this study were more willing to embrace change. However, it is important to highlight that the interviewed nurses belonged to CHC centers in two different regions, representing both urban and rural parts of Sweden, and in both high- and low-socioeconomic areas, which adds to the generalizability of the results to the rest of Sweden. The study population were all female with a broad range in years of experience within CHC, making them representative of the profession at large, as almost 90% of all licensed nurses are women [31]. It is also important to mention that the findings and the themes were created by a research team with similar skillsets. This might have affected how the codes and themes were created. To minimize such risk, there was always a continuous discussion about the perceived findings and themes within the research group. Finally, there is a need to further investigate the perspectives of CHC nurses regarding food introduction, physical activity/active play, and screen time with parents, focusing particularly on how these issues are related to parental needs and limited parental support networks. Future studies are also needed to evaluate how CHC nurses cope and manage with discussing sensitive topics such as excess screen time with parents, finding sufficient time to discuss important topics during individual scheduled appointments as well as how to better engage parents from other cultures.

## 5. Conclusions

This study found that CHC nurses have seen an increase in the need for support among today’s parents. Time, the need to tailor information, and confidence to address sensitive topics were perceived as the largest barriers in daily work for the nurses. Furthermore, this study also displayed large variations in the existing CHC nurse-run parental groups in terms of structure, content, and frequency. Overall, the CHC nurses displayed a willingness and openness to change and to develop current working methods and were positive about the implementation of a program such as INFANT. The implementation of such a program has the potential to ease the workload of the nurses and at the same time, contribute to greater equality in care for parents and children across Sweden.

## Data Availability

The anonymized transcribed interviews are available from the corresponding author upon reasonable request.

## Supplementary Materials

The following are available online at https://www.mdpi.com/article/10.3390/children8080696/s1, File S1: Consolidated criteria for reporting qualitative research (COREQ) checklist, File S2: Core questions asked to the participants.
