# Supplementary material for: How to Support Child Healthcare Nurses in Sweden to Promote Healthy Lifestyle Behaviors from the Start of Life
Source: Children (Basel). 2021 Aug 12;8(8):696. doi: 10.3390/children8080696 (PMC8392879; doi:10.3390/children8080696)
Supplement: Supplementary file 1 [file children-08-00696-s001.zip › File S2.pdf]

Interview guide

1. When were you born? (year and month)

2. How long have you been professionally active as a child healthcare (CHC) nurse?

3. If you think about all the parents you have met and are meeting in your work, how do you experience the parents' need for information and support during the first 18 months?

4. Do you feel that the parents come to you at the CHC center with their questions or are they searching for answers elsewhere?

5. After reading parts of the National Handbook (Rikshandboken), I am impressed by the amount of information that should be conveyed to the parents. When we interviewed parents in a study last year, it was clear that it was very different in how much the parents themselves asked for information; some said they used to ask a lot during their visits at CHC while others did not really dare, or they forgot what they wanted to ask, or they simply did not know what to ask about.

   - How would you describe working with the balance of providing information to parents and answering parents' questions?
   - Do you feel that you have the time to answer all the questions during your meetings with the families?
   - What can be challenging in these situations?

6. If you think about the first 18 months, can you tell me a little about how you work within your CHC center. What working methods and special programs are available within the CHC to work with lifestyle behaviors and to support parents in establishing good eating and exercise habits for their children (e.g., through breastfeeding/bottle feeding, food introduction, healthy food, screen time and active play)?

7. Do you offer parent groups and if so, how are these organized?

8. Regarding the parent groups, have you been trained in leading group discussions?

9. How confident do you feel in your role in giving parents advice and answers to thoughts and questions about:
   - Food introduction, healthy food and feeding?
   - Screen time?
   - Physical activity/active play for the children?

10. How do you think the need of information and support differs between Swedish-born parents and parents of other origins or other cultural backgrounds?

11. We collaborate with researchers from Australia where they work with the target group parents of children aged 0-1.5 years and offer extra information and support about lifestyles in different ways. One of the programs include e.g., six different educational workshops given within parent groups at CHC during the time when the children are 2–18 months old. These sessions cover the topics: feeding, food introduction, active play, screen time and other aspects of the child's diet and movement. The group sessions are also supplemented by a mobile app with the same type of information, which the parents have access to.

    - What do you think about such a program for parents of young children here in Sweden?
    - What advantages and disadvantages do you see in offering this extra type of support within parent groups within CHC?
    - Since it is not possible to meet in person today due to the current situation, these workshops, as I mentioned, are now held digitally. How do you feel about holding group meetings/workshops for parents digitally as opposed to physically at CHC, even post Covid-19? What advantages and disadvantages can you see? Do you think you can reach out to more parents with digital meetings?

- What advantages and difficulties do you see in offering an app as an extra support in your health promotion work within CHC?
- What would need to be changed within your centre for you and your colleagues to be able to implement this type of program within your routine working methods within CHC?

12. We envision that the parents would be invited to the first workshop when the child is 2 months old, with a total of 6 workshops until the child is 18 months old. Do you think this is a suitable time?

13. What do you think is particularly important information or type of support that should be included in such a program in order for you to use it or to recommend it to "your" families?

14. What do you think is important for us to consider in the design of such a program so that it can be implemented within the routine care within CHC?

15. What do you think is important to keep in mind for such a program/support to be able to reach out and to work for different types of parent groups (e.g., foreign-born, different socio-economic or cultural backgrounds)?

16. What wishes and expectations do you have as a CHC nurse for a new program supporting parents in breastfeeding as well as healthy eating and physical activity habits for their children?

17. What other thoughts, opinions or ideas would you like to bring up before we end this interview that we may have missed talking about?
